# Cromolyn, a New Hope for Limited Treatment of Neutrophilic Asthma: a Phase II Randomized Clinical Trial

**Published:** 2019-03

**Authors:** Majid Mirsadraee, Zahra Sabbagh Sajadieh, Shadi Ghafari, Afsaneh Tavakoli, Saeedeh Sabbagh Sajadieh

**Affiliations:** 1 Department of Internal Medicine, Faculty of Medicine, Islamic Azad University, Mashhad Branch, Mashhad, Iran,; 2 Department of Internal Medicine, Golestan University of Medical Sciences, Sayyad Shirazi Hospital, Gorgan, Iran; 3 Shahid Hashemi Nezhad Research Center, Ministry of Education, Mashhad, Iran,; 4 Independent Pharmacist, Mashhad, Iran,; 5 Department of Pathology, Taleghani Medical Institute and Research Center, Mashhad University of Medical Sciences, Mashhad, Iran.

**Keywords:** Cough, Cromolyn, Disodium cromoglycate, Asthma, Resistant asthma

## Abstract

**Background:**

In this study, we aimed to determine the effects of cromolyn on the clinical outcomes and neutrophilic inflammation in patients with resistant cough-variant asthma.

**Materials and Methods:**

Patients with cough-variant asthma, with normal physical examination and spirometry results, were treated by inhaled corticosteroids, antileukotrienes, antibiotics, and proton-pump inhibitors according to the Global Initiative for Asthma (GINA) guidelines. Seventy patients, who were resistant to these treatments, were enrolled in this double-blind randomized clinical trial. After randomization, eligible subjects received a cromolyn metered dose inhaler (MDI) or a placebo MDI, which was completely similar in appearance to the cromolyn inhaler. The primary outcomes included cough and Asthma Control Test (ACT) score.

**Results:**

Based on the findings, cough significantly decreased with cromolyn therapy, compared to the placebo group. Other clinical findings, including dyspnea, sputum production, and nocturnal symptoms, also improved. The ACT score significantly improved to a nearly normal level (23.53±2.25) in the cromolyn group. Moreover, fractional exhaled nitric oxide (FeNO) significantly decreased with cromolyn treatment (14±9.31 ppm after treatment vs. 28.88±27.39 before treatment). The neutrophil count significantly decreased in the cromolyn group (from 44±24.2% before the trial to 34.08±16.7% after the trial), while it increased in the placebo group (from 39.67±26.47% to 56.71±27.22%).

**Conclusion:**

Cromolyn improved the clinical findings of resistant cough-variant asthma and could suppress neutrophilic inflammation.

## INTRODUCTION

Cough-variant asthma is a type of asthma, characterized by cough without dyspnea, wheezing, or spirometry derangement (1,2). Treatment of this disease is similar to routine asthma (3). Occasionally, physicians encounter asthmatic patients who are resistant to both inhaled corticosteroids (ICS) and antileukotrienes (4).

Further investigation for lung infection and treatment of obscure gastroesophageal reflux disease (GERD) are commonly recommended for these patients (5). The predominant inflammatory cells in cough-variant asthma are eosinophils, which are associated with high fractional exhaled nitric oxide (FeNO) and good response to ICS (6).

Many asthmatic patients are resistant to ICS and may show neutrophil predominance in the submucosa. Some of the well-known mediators in the pathogenesis of neutrophilic asthma are as follow interferon gamma (IFN-γ), interleukin-8 (IL-8), IL-11β, and IL-17. (7,8). Neutrophilic asthma is not considered a very rare condition. In a previous study, non-eosinophilic asthma was reported in 47% of patients with mild to moderate asthma, who were not responsive to ICS (9), while neutrophilic asthma (more than 76% of sputum inflammatory cells) was reported in 19% of the subjects (10). Neutrophilic asthma tends to be more resistant to ICS and persists for longer periods (11). A recent algorithmic approach to severe asthma considered the treatment of neutrophilic asthma as a clinical problem with no established therapy (12).

Cromolyn (disodium cromoglycate) and nedocromil are two medications with few side effects, which are potential therapeutic substitutes for the resistant form of cough-variant asthma. Although use of these two drugs is no longer recommended in the Global Initiative for Asthma (GINA) guidelines (7,8), they have been recommended in some valid references (13). Moreover, cromolyn has shown good results in children (14). This medication can prevent house-dust induced bronchospasms (15) and effectively relieve exercise-induced asthma (16).

Experimental studies on the anti-inflammatory effects of chromones have shown that they reduce the accumulation of neutrophils after the induction of animal models by ovalbumin (17). Moreover, a study on bronchial mucosa obtained from nine asthmatic patients by bronchoscopy showed the effective reduction of inflammatory cells, including eosinophils, neutrophils, lymphocytes, macrophages, and adhesion molecules by cromolyn (18). However, some questions arise as to whether cromolyn can manage resistant asthma better than standard therapy and what effects cromolyn has on neutrophilic asthma. The primary goal of this study was to evaluate the effects of cromolyn on cough-variant asthma and to determine the changes in neutrophilic inflammation.

## MATERIALS AND METHODS

This study was performed during 2011–2013 and consisted of two phases: 1) screening and pretrial treatment (one year); and 2) a clinical trial with cromolyn and placebo (one year).

### Screening Period and Pretrial Phase:

A total of 421 subjects (212 females and 209 males), aged above 15 years, who were referred to a pulmonary subspecialty clinic, were selected to participate in the pretrial phase. The subjects met the following criteria: 1) coughing for more than three weeks without a significant history of dyspnea or wheezing; 2) history of intermittent asthma and its exacerbation; 3) history of hyper-responsiveness based on the patient’s report; 4) no recent history of respiratory tract infection, marked heartburn, or regurgitation; 5) normal spirometry including bronchodilator challenge; and 6) normal chest X-ray and paranasal sinus radiogram in the event of postnasal drip. Subjects who met these criteria were treated with ICS (fluticasone or budesonide), montelukast tablets, high-dose proton-pump inhibitors (PPI), and oral antibiotics (in case of respiratory infection), using the stepwise approach. If coughing persisted, the patient was enrolled in the clinical trial as a candidate for treatment with cromolyn inhalation.

### Clinical Trial

***Groups and random allocation:*** The participants were randomly divided into two groups. One group received cromolyn inhalers (MDI, Cromolex®, Sina Darou, Tehran, Iran), while the second group received placebo inhalers, which were completely similar to the Cromolex inhalers. Each puff contained 1 mg of cromolyn. Each subject took two puffs four times a day for 40 days using an inhaler, which was used with a spacer. Albuterol MDI was also prescribed to both groups for use as a rescue medicine, if needed. It should be noted that all previous drugs were discontinued.

***Blinding:*** Both the drug and placebo were coded by a pharmacist and prescribed by another pharmacist, who was blinded to the codes of drugs and placebo. Patients and the treating physician were unaware of group assignments throughout the study. The outcome variables were evaluated by physicians and technicians, who were blinded to the study groups.

### Phase II Clinical Trial

***Outcome variables:*** The primary endpoints included cough improvement and score of Asthma Control Test (ACT), which is a valid questionnaire for evaluating asthma (19). The secondary endpoints were improvement of dyspnea and detection of significant changes in forced expiratory volume-one second (FEV1), FEV1/forced vital capacity (FVC), FeNO, and inflammatory cells in the sputum. Spirometry was carried out using a turbine spirometry device (Superspiro, Micomedical Co., London, UK), according to the American Thoracic Society/European Respiratory Society (ATS/ERS) guidelines (20). FeNO was measured using NObreath device (Bedfont Medical Instruments, London, England).

***Sputum induction:*** Sputum was induced by 5% saline inhalation. The subjects were premedicated with two puffs (100 μg per puff) using a salbutamol inhaler, and inhalation was done by a compressor-type nebulizer (CX3, Omron, Japan), according to the ERS guidelines (21).

***Sputum processing:*** A liquid-based commercial kit (E-Prep Plus Sol, Tehran, Iran) was used for sputum preparation. For cell type identification, two unfixed sputum smears were prepared, and the mean results of the two slides were recoded. For classification of inflammatory cells, subjects with an eosinophilic percentage above 3% were classified as eosinophilic, those with a neutrophilic percentage above 76% were classified as neutrophilic, and those with both conditions were classified as the mixed type; patients with none of these conditions were classified as paucigranulocytic (10,22).

***Follow-up:*** Phone calls were made to follow-up the patients every two weeks. Any participant who complained of irritating coughing was eliminated from the study and received other treatments.

***Ethical considerations:*** This clinical trial was registered in the Iranian Registry of Clinical Trials (registration number: IRCT201108042695N3). Informed consent was obtained from all participants, and the benefits, side effects, and course of treatment were discussed. This study was approved by the university’s institutional review board.

### Statistical Analysis

Comparison of the outcomes of treatment between the cromolyn and placebo groups was performed, using Chi-square test, Fisher’s exact test, Mann-Whitney U test, and student’s t-test. The outcomes of treatment after the trial were analyzed by McNemar’s test, ANCOVA, paired t-test, and Wilcoxon signed-rank test. SPSS version 19 was used for statistical analysis, and the level of significance was set at 0.05 (see the supplementary file for more details).

## RESULTS

### Pretrial Phase

The mean age of the subjects was 43±2 years (range: 7–77 years), and no significant difference was found between males and females (t=0.4, P=0.68). The majority of the participants were housewives (35%) and clerks (22.3%). None of the subjects reported exposure to heavy air pollution. Comparison of clinical findings between the two groups showed insignificant differences, except for dyspnea, which was more predominant in the cromolyn group (Table 1).

**Table 1.** Comparison of demographic, clinical, physiological, and cytological findings in subjects enrolled in the trial for treatment of resistant cough variant of asthma using the cromolyn inhaler.

	**Total**	**Before trial**		**After trial**	

		**Cromolyn**	**Placebo**	**Cromolyn**	**Placebo**
**Female/Male**	24/24	14/20	10/4	14/20	10/4
**Age (Years)**	43±17	43±15	43±19	43±15	43±19
**Cough**	48 (100%)	34 (100%)	14 (100%)	4 (12%)^*†^	12 (75%)
**Dyspnea**	34 (70%)	27 (80%)	7 (50%)	4 (12%)^*†^	7 (50%)
**Sputum**	41 (85%)	29 (85%)	12 (85%)	7 (20%)^*†^	10 (71%)
**Nocturnal symptoms**	30 (62%)	23 (67%)	7 (50%)	2 (6%)^*†^	4 (28%)
**AHR**	36 (75%)	27 (79%)	9 (64%)	10 (29%)^†^	8 (57%)
**PND**	21 (43%)	14 (41%)	7 (50%)	4 (12%)^*†^	10 (71%)
**FEF25-75/FVC**	1.11±0.26	1.14±0.28	1.07±0.23	1.17±0.31	1.11±0.24
**FENO (PPM)**	30.3±33.3	28.88±27.39	30.93±44.14	14±9.31^*†^	30.69±39
**ACT**	15.1±3.5	14.97±3.97	14.86±2.97	23.53±2.25^*†^	18.07±3.43^‡^

AHR= Airway hyper-responsiveness, GERD= Gastero-esophageal reflux, PND= Post-nasal drip, FENO= Fraction of exhaled nitric oxide, ACT= asthma control test

* = Significant difference after the trial in the cromolyn group

† = Significant difference between the cromolyn and placebo after the trial

‡ = Significant difference after the trial in the placebo group

### Trial

Thirty-four subjects in the cromolyn group and 14 subjects in the placebo group completed the study. One subject from the cromolyn group and 21 subjects from the placebo group complained of chronic cough, which led them to leave the study and discontinue treatment. The clinical findings significantly improved in the cromolyn group (Table 1), except airway hyper-responsiveness. The ACT results showed an improvement in asthma to almost normal levels (mean ACT score after the trial: 23.53±2.25). The clinical findings did not show any significant changes in the placebo group, while the ACT score improved significantly (Table 1). However, improvement of ACT score in the cromolyn group was significantly higher than the placebo group. The frequency of all clinical findings was significantly lower in the cromolyn group, compared to the placebo group (Table 1).

The spirometric parameters did not significantly change in the cromolyn and placebo groups after the trial, except for FEV1/FVC (Table 1). FeNO was mildly elevated in both groups. After the trial, it significantly decreased in the cromolyn group, while no significant change was reported in the placebo group (Table 1).

**Figure 1.**
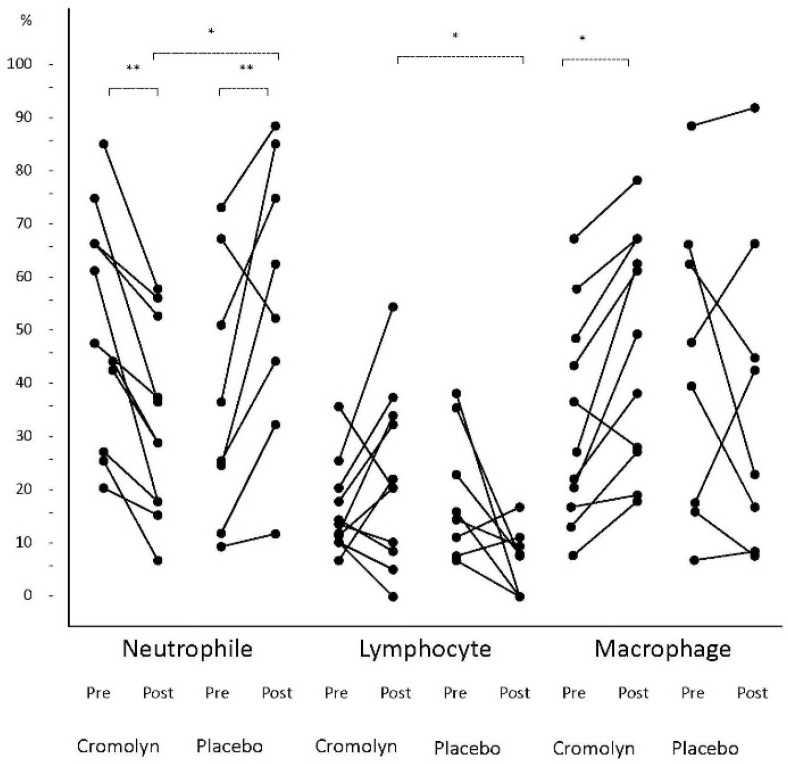
Comparison of percentage of sputum inflammatory cells (eosinophil not included) in subjects suffering from cough variant of asthma treated with cromolyn and the placebo.

***Inflammatory cells:*** Based on the findings, 17% of the subjects in the pretrial phase and 65% of the subjects in the post-trial phase were unable to produce enough sputum for cytological analysis (Table 2). The most predominant inflammatory cells in the cromolyn group were neutrophils (44±24.2%), which significantly decreased to 34.08±16.7%, with the mean decrement of 18±5.9% after the trial (paired t-test= 3.19; P=0.008) (Figure 1). As a compensatory mechanism, the level of macrophages increased from 39.37±25.07% to 47.92±22.58% (t-test=−4.4; P=0.001). Lymphocytes and eosinophils did not show significant changes (15±9.63% vs. 17.42±12.2% for lymphocytes and 0.52±1.76% vs. 0.33±0.88% for eosinophils). In the placebo group, the neutrophil percentage increased significantly from 39.67±26.47% to 56.71±27.22% (t=−2.5, P=0.02) (Figure 1).

**Table 2.** Comparison of cytological classification between cough variant of asthma subjects treated with the cromolyn inhaler

	**Before trial**		**After trial**	

	**Cromolyn**	**Placebo**	**Cromolyn**	**Placebo**
**Eosinophilic**	0	1 (7%)	0	0
**Neutrophilic**	5 (14%)	0	0^*†^	3 (21%)
**Paucigranulocytic**	22 (67%)	11 (78%)	12 (35%)^*†^	4 (28.6%)
**No sputum**	7 (21%)	2 (17%)	22 (65%)^*†^	7 (50%)
**Total**	34	14	34	14

* = Significant difference after the trial in the cromolyn group

† = Significant difference between the cromolyn and placebo after the trial

Comparison of inflammatory cells between the cromolyn and placebo groups after the trial showed that the neutrophil percentage was significantly lower in the cromolyn group, compared to the placebo group (34.08±16.7% vs. 56.71±27.22%; t=−2.56, P=0.02). On the contrary, the lymphocyte percentage was significantly higher in the cromolyn group, compared to the placebo group (17.42±12.2% vs. 7.14±4.52%; t=2.12, P=0.04) (Figure 1). The macrophage and eosinophil percentages were higher in the cromolyn group, but the difference was not significant (17.42±12.2% vs. 7.14±4.52% for lymphocytes and 0.33±0.88% vs. 0.29±0.75% for eosinophils) (Figure 1).

***Cytological classification:*** The eosinophilic pattern (eosinophil >3%) was observed in one subject from the placebo group, who was classified as neutrophilic after the trial. The neutrophilic pattern (>76%) was found in 5 (14%) subjects from the cromolyn group. After the trial, two subjects were classified as paucigranulocytic, and three subjects did not have enough sputum for sputum induction (Table 2). Overall, the neutrophilic pattern disappeared in the cromolyn group, while it increased in the placebo group; the difference was significant between the groups (Fisher’s exact test=0.01). The paucigranulocytic pattern was the predominant pattern in both groups, but its frequency decreased significantly in the cromolyn group due to increased sputum production. On the other hand, in the placebo group, the paucigranulocytic pattern decreased, as the neutrophilic pattern was observed in two subjects.

## DISCUSSION

In the pretrial phase, subjects with cough-variant asthma were treated with ICS, anti-leukotrienes, PPIs, and antibiotics. Next, subjects who still complained of coughing were enrolled in a double-blinded randomized clinical trial and received cromolyn inhalers. The results showed that cromolyn could successfully decrease coughing and elevate the ACT score to a nearly normal level. It also improved other respiratory symptoms, including dyspnea, sputum production, night symptoms, and exacerbations, and suppressed FeNO. Resolution of symptoms was not spontaneous, as the condition of none of the subjects in the placebo group improved naturally. The frequency of presentations was not significantly different in four seasons. Also, considering the long period of the pretrial phase, the temporary effects of air pollution on the disease activity were ruled out.

Neutrophil was the main inflammatory cell in the selected groups during the trial. Cromolyn could decrease inflammation in most of the subjects, as indicated by the reduction in neutrophil percentage and FeNO. On the contrary, inflammation increased in the placebo group. Presence of bacteria was confirmed by bacterial culture in non-bronchiectatic asthmatic subjects (23). In this regard, a previous study reported that *Haemophilus influenzae*, *Pseudomonas aeruginosa*, and *Staphylococcus aureus* had direct relationships with the duration of asthma. Moreover, an experimental study showed that *H. influenzae* infection, associated with allergic airway disease, resulted in T helper 17 cell reactions (5). These reactions induced neutrophilic inflammation without manifestations of active infection. Consequently, antibiotic therapy may improve the outcomes of asthma therapy.

A previous study used clarithromycin as an antibiotic and anti-inflammatory agent for resistant and neutrophilic asthma (24). Although clarithromycin had beneficial effects, we believe that cromolyn is more effective and has fewer side effects. In fact, the frequency of side effects was 12% in clarithromycin treatment and 3% in cromolyn treatment according to our study. It seems that these antibiotic therapies are suitable for more severe cases of asthma, while in patients with cough-variant asthma, it is suggested to treat the patients with cromolyn as a medication without side-effects.

FeNO, as a marker of inflammation, is a useful tool for asthma (6). In a previous study, FeNO in cough-variant asthma was lower than typical asthma (23). In the present study, the cut-off point of FeNO was 28 ppm. Detection of neutrophilic asthma is made by sputum analysis. In our experience, persistent and resistant asthma is the best predictive clinical marker for neutrophilic asthma. However, the mechanism of action of cromolyn in neutrophilic inflammation is not fully understood. Mechanisms, such as inhibition of NADPH oxidase and oxygen production in neutrophils (25), inhibition of neutrophil chemotaxis (14), and cell rolling, velocity, adhesion, and migration (26) by reducing intracellular free calcium levels (27) have been introduced.

## CONCLUSION

In conclusion, cromolyn could relieve coughing and other symptoms of resistant asthma. It seems that cromolyn is the second most effective drug for neutrophilic asthma after clarithromycin.
